# Psychometric evaluation of the translated arabic version of the geriatrics health behavior questionnaire (GHBQ) for geriatric nurses: a cross-sectional study

**DOI:** 10.1186/s12912-024-02164-9

**Published:** 2024-08-13

**Authors:** Mostafa shaban, Huda Hamdy Mohammed, Fatma Gomaa Mohamed Amer, Hla Hossni Elsayed, Sayed Ibrahim Ali, Ateya Megahed Ibrahim

**Affiliations:** 1 Faculty of Nursing, Cairo University, Cairo, Egypt; 2 Educational Psychology Department, College of Education, Helwan University, Cairo, Egypt; 3 Family and Community Health Nursing Department, Faculty of Nursing, Port Said University, Port Said, Egypt; 4 Nursing College, Prince Sattam Bin Abdualziz University, Alkarj, KSA Saudi Arabia

**Keywords:** Geriatrics, Health behavior, Psychometric evaluation, Arabic population, Cultural adaptation

## Abstract

**Background:**

The Geriatrics Health Behavior Questionnaire (GHBQ) is essential for assessing health-related behaviors among older adults populations. This study focuses on the translation, cultural adaptation, and psychometric evaluation of the Arabic version of the GHBQ to ensure its relevance and accuracy for Arabic-speaking older adults individuals.

**Methods:**

This cross-sectional study was conducted at the Cairo University Educational Hospital’s outpatient clinic. The GHBQ was translated and culturally adapted through a systematic process, including initial translation, back-translation, expert review, and pilot testing. The psychometric properties of the Arabic-translated GHBQ were evaluated using a sample of 200 older adults Arabic-speaking participants. Reliability was assessed using Cronbach’s alpha (α) and Intraclass Correlation Coefficient (ICC). Validity was evaluated through Content Validity Index (CVI), Exploratory Factor Analysis (EFA), and Confirmatory Factor Analysis (CFA).

**Results:**

The Arabic GHBQ demonstrated excellent reliability with Cronbach’s alpha values ranging from 0.74 to 0.87 across subscales and ICC values confirming reproducibility (ICC = 0.82). The CVI indicated strong content validity (average CVI = 0.91). EFA revealed a five-factor structure, explaining 72% of the variance, with all factor loadings exceeding 0.60. CFA supported the questionnaire’s structure with fit indices meeting recommended criteria: χ²/df = 2.05, NFI = 0.92, TLI = 0.94, GFI = 0.90, SRMR = 0.05, AIC = 140.35, and BIC = 160.22. Criterion validity was confirmed through significant correlations with established health behavior measures (*r* = 0.63, *p* < 0.001).

**Conclusions:**

The culturally adapted Arabic version of the GHBQ is a reliable and valid tool for assessing health behaviors in the older adults population in Egypt. This instrument can aid healthcare providers in identifying and addressing health behaviors, ultimately improving the well-being of this demographic. Future research should focus on expanding the sample and comparing the GHBQ with other similar tools used in Arabic-speaking populations.

**Supplementary Information:**

The online version contains supplementary material available at 10.1186/s12912-024-02164-9.

## Introduction

The worldwide demographic transition towards a population that is increasingly older poses substantial problems and possibilities for public health and socioeconomic systems. By 2020, the population of individuals aged 60 and beyond exceeded that of children under the age of five [1]. Furthermore, it is projected that by 2030, one out of every six people globally will be over the age of 60. By the year 2050, it is anticipated that this figure would increase by more than two times, reaching a total of 2.1 billion [2]. Additionally, the population of those aged 80 and beyond is forecast to increase fourfold, reaching a total of 426 million. The rate of growth is particularly significant in low- and middle-income nations, where 80% of the old population will live [3, 4].

Aging is an intricate phenomenon that occurs as a consequence of the gradual buildup of molecular and cellular harm over time [5, 6]. This leads to a reduction in both physical and mental capacities, as well as an increased vulnerability to diseases. Typical health problems linked to getting older include auditory impairment, clouding of the lens in the eye, degenerative joint disease, high blood sugar levels, low mood, and cognitive decline. In addition, older adults individuals frequently encounter weakness, urine incontinence, falls, cognitive impairment, and pressure ulcers [7, 8].

Gaining insight into the health habits of older persons is essential, as these activities have a substantial influence on their general state of health, contentment, life fulfillment, and ability to bounce back from adversity [9–11]. Studies suggest that psychological characteristics, such as wisdom and a positive outlook, play a crucial role in assisting older individuals in managing stress and overcoming developmental obstacles [12]. Older adults who demonstrate wisdom actively participate in constructive social relationships and uphold a strong feeling of self-confidence, significance, and direction [13, 14]. These characteristics are associated with emotional well-being and the promotion of happy experiences while reducing negative ones. Research has demonstrated that life satisfaction, which is a part of positive orientation, affects the connection between personal resources and health habits among older individuals. Increased life satisfaction can serve as a driving force for individuals to strive for improved health outcomes and uphold a healthier way of life [15–17].

The Geriatrics Health Behavior Questionnaire (GHBQ) was developed to provide a comprehensive tool for assessing health behaviors in the older adults. This instrument was designed to evaluate various behaviors including diet, physical activity, social engagement, and adherence to medical advice, with the aim of identifying areas for intervention to improve health outcomes in the aging population [18].

The translation and cultural adaptation of health behavior questionnaires for Arabic-speaking communities is essential since there are considerable cultural and linguistic disparities that impact health behaviors and perceptions [19, 20]. Cultural adaptation goes beyond mere translation; it entails adjusting the material to conform to the particular cultural norms, values, beliefs, and rituals of Arabic-speaking communities [21]. This procedure guarantees that the questionnaire is both linguistically precise and culturally appropriate, effectively connecting with the intended group. As a result, it improves the dependability and accuracy of the obtained data [22].

The objective of this study is to comprehensively assess the psychometric characteristics of the Arabic-translated Geriatrics Health Behavior Questionnaire (GHBQ) in the specific setting of Egypt. The study’s significance rests in its capacity to offer a validated instrument for precisely evaluating and examining the health habits of the older adults in Egypt, a nation with distinct cultural and healthcare attributes. This tool is essential for the development of specific health interventions and policies, enhancing the standard of care for older adults individuals, and promoting research in the field of aging and health in the Arabic-speaking region. The effective adaptation and validation of the GHBQ in Egypt could serve as a paradigm for analogous adaptations in other Arabic-speaking nations.

## Method

### Study design

This research employed a cross-sectional study design to evaluate the psychometric properties of the Arabic-translated Geriatrics Health Behavior Questionnaire (GHBQ). Conducted at the outpatient clinic of Cairo University Educational Hospital, this approach was chosen for its efficiency in assessing the prevalence and distribution of health-related behaviors and attitudes among the older adults population at a specific point in time. the study was conducted between November 2023 to January 2024.

Participants were surveyed once, providing a comprehensive snapshot of their health behaviors and attitudes. The focus was on assessing the reliability and validity of the GHBQ in an Arabic-speaking context. Reliability was examined through measures such as internal consistency and Cronbach alpha. Validity was explored through content, construct, and criterion validity assessments. This method provided essential insights into the effectiveness of the translated GHBQ in capturing relevant health behaviors and attitudes of the older adults in the Egyptian context.

### Setting

The study was conducted at the outpatient clinic of Cairo University Educational Hospital, located in Cairo, Egypt. This facility was chosen for its significant role as a major healthcare provider for the older adults population in the region. The outpatient clinic is known for its diverse patient demographic, making it an ideal location for gathering a representative sample of the older adults Arabic-speaking population. The clinic’s setting also facilitated convenient access to participants and provided an appropriate environment for administering the questionnaire in a real-world clinical context.

### Participants

Participants were older adults individuals aged 60 and above attending the outpatient clinic of Cairo University Educational Hospital. Inclusion criteria included Arabic-speaking patients capable of providing informed consent and without cognitive impairments that could affect questionnaire comprehension. The Mini-Mental State Examination (MMSE) was used to assess cognitive status before obtaining consent, with a cut-off score for the Egyptian version of the MMSE set at 23. Participants scoring below this threshold were excluded from the study. In total, 18 participants were excluded based on cognitive assessment. Exclusion criteria also comprised individuals with severe hearing, speech, or cognitive impairments that could impede effective communication. This approach ensured a sample representative of the general older adults population in the clinic, allowing for a comprehensive evaluation of the GHBQ’s applicability in this demographic.

### Sampling technique and sample size

A convenience sampling method was initially used to recruit participants who were readily available during their visits to the outpatient clinic. To enhance representativeness, the sample was later stratified by gender, educational level, and geographic area (urban vs. rural), ensuring a broader demographic distribution.

To determine the appropriate sample size, a power analysis was conducted. A commonly accepted guideline for factor analysis is to have at least 5 to 10 participants per item in the questionnaire [23, 24]. For the GHBQ, which comprises 17 items, this would suggest a minimum sample size of 85 to 170 participants. However, to enhance the robustness of the analysis, it is often recommended to exceed this minimum threshold. A sample size of 200 provides a participant-to-item ratio of approximately 12:1, which is well within and exceeds these guidelines, thereby improving the reliability and generalizability of the findings.

### Translation and cultural adaptation process

The translation and cultural adaptation of the GHBQ involved a systematic and multi-phased approach:

#### Initial translation

The questionnaire was first translated into Arabic by a team of bilingual health professionals experienced in geriatrics and public health. This team ensured the translation retained the original meaning while being linguistically appropriate.

#### Back-translation

A separate team, unaware of the original questionnaire, back-translated the Arabic version into the source language. This step was crucial to identify and rectify any discrepancies or misinterpretations in the translation.

#### Expert review panel

The translated version was then reviewed by an expert panel comprising geriatricians, psychologists, linguists, and cultural experts. This panel assessed the translation for accuracy, relevance, and cultural appropriateness, ensuring that the questionnaire resonated with the cultural nuances, health beliefs, and practices of the Egyptian older adults population.

#### Pilot testing and focus groups

The preliminary Arabic version was pilot tested with a small group of older adults individuals to check for clarity and comprehension. Focus groups were conducted to gather feedback on the questionnaire’s understandability, relevance, and cultural appropriateness. These discussions helped in identifying any culturally sensitive issues and in understanding the participants’ perspectives on health behaviors.

#### Revisions based on feedback

Based on the feedback from the pilot testing and focus groups, necessary revisions were made to the questionnaire. This iterative process ensured that the final Arabic version of the GHBQ was not only a linguistic translation but also a culturally adapted tool suitable for the target population.

#### Final expert approval

The final version of the questionnaire received approval from the expert panel, confirming its suitability for use in the intended population.

This thorough translation and cultural adaptation process aimed to ensure that the Arabic version of the GHBQ was both linguistically accurate and culturally sensitive, thereby enhancing its validity and reliability in the context of the Egyptian older adults population.

### Data collection tools

#### Arabic version of the GHBQ

The primary tool for data collection is the Arabic-translated Geriatrics Health Behavior Questionnaire, adapted for cultural relevance and comprehension.

#### The geriatrics health behavior questionnaire (GHBQ) includes 17 items across seven dimensions

Physical activity, nutrition status, medication adherence, stress management, smoking and alcohol consumption, sleep quality, and medical check-ups. The scoring of GHBQ items ranges from 0 to 1 based on the accumulation approach. The total questionnaire scores range from 0 to 17, with higher values indicating better health behaviors.

#### Demographic and health information form

A form to collect basic demographic information (age, gender, education level, etc.) and general health information (medical history, medication use, etc.) from participants.

#### Mini-mental state examination (MMSE)

To ensure participants’ cognitive ability to understand and respond to the questionnaire. The Mini-Mental State Examination (MMSE) is a widely used cognitive assessment tool in clinical settings and research. It evaluates various aspects of cognitive function, including orientation to time and place, immediate and delayed recall, attention and calculation, language abilities, and visual construction. The MMSE is scored on a scale of 0 to 30, with higher scores indicating better cognitive function.

### Data collection procedures

#### Participant recruitment and informed consent

The recruitment process was conducted at the outpatient clinic of Cairo University Educational Hospital. Potential participants, older adults individuals aged 60 and above, were approached and briefed about the study. They received detailed information regarding the study’s purpose, procedures, and their rights, including confidentiality and voluntary participation. Written informed consent was obtained from each participant who agreed to partake in the study.

#### Administration of the GHBQ

The Arabic version of the Geriatrics Health Behavior Questionnaire was administered in a comfortable and private setting within the clinic. Participants had the option to complete the questionnaire independently or with assistance from a researcher. The environment was designed to ensure privacy and encourage honest and accurate responses.

#### Collection of demographic and health information

Participants also completed a demographic and health information form. This form collected essential data such as age, gender, educational background, and medical history. The purpose of this additional data collection was to provide context for the responses on the GHBQ and to enable a more comprehensive analysis of the results.

#### Data recording and management

All responses were meticulously recorded and entered into a secure database. Participants were assigned unique identifier codes to maintain confidentiality. The data, both in paper and electronic forms, were stored securely, with restricted access to ensure privacy and data integrity.

### Data analysis

Statistical analyses were conducted using SPSS (Statistical Package for the Social Sciences) version 25 and AMOS (Analysis of Moment Structures) version 25 to evaluate the psychometric properties of the Arabic-translated Geriatrics Health Behavior Questionnaire (GHBQ). Descriptive statistics, including means, standard deviations, frequencies, and percentages, were used to summarize demographic characteristics and responses to the GHBQ items.

For reliability analysis, internal consistency was assessed using Cronbach’s alpha for the overall questionnaire and each subscale, with a threshold value of α ≥ 0.70 considered acceptable. Test-retest reliability was evaluated using the Intraclass Correlation Coefficient (ICC) in a subset of participants who completed the questionnaire twice within a two-week interval. Content validity was assessed by a panel of experts in geriatric health and questionnaire development using the Content Validity Index (CVI), where item-level and scale-level CVI scores were calculated, with a threshold of 0.80 considered acceptable.

Construct validity was examined through both Exploratory Factor Analysis (EFA) and Confirmatory Factor Analysis (CFA). EFA was performed using Principal Component Analysis (PCA) with Varimax rotation in SPSS, retaining factors with eigenvalues greater than 1 and factor loadings of 0.40 or higher. The suitability of the data for factor analysis was confirmed using the Kaiser-Meyer-Olkin (KMO) measure of sampling adequacy and Bartlett’s Test of Sphericity. CFA was conducted in AMOS to test the fit of the factor structure identified by EFA, using maximum likelihood estimation. Model fit was evaluated using several indices: Comparative Fit Index (CFI), Tucker-Lewis Index (TLI), Root Mean Square Error of Approximation (RMSEA), and Standardized Root Mean Square Residual (SRMR). Good fit was indicated by CFI and TLI values of 0.90 or higher, RMSEA values of 0.08 or lower, and SRMR values of 0.08 or lower.

Criterion validity was assessed by conducting correlation analyses between the GHBQ scores and relevant external measures, such as existing health behavior scales, using SPSS. Significant correlations (*p* < 0.05) with these measures confirmed the questionnaire’s effectiveness in capturing the intended constructs.

To identify predictors of health behaviors among the older adults, multiple regression analysis was performed with GHBQ scores as the dependent variable and demographic characteristics (age, gender, education, marital status, living arrangement) as independent variables. This analysis was conducted in SPSS, and both univariate and multivariate analyses were combined into a single regression table, presenting coefficients, standard errors, and p-values. The assumptions of normality, linearity, homogeneity, and homoscedasticity were checked for any violations throughout the analysis.

### Ethical consideration

All methods in the present study were carried out in accordance with relevant guidelines and regulations. Ethical approval for the study was obtained from the ethics committee of the Faculty of Nursing/Port Said University with Code number NUR (5/11/2023) (31). Also Informed consent to participate was obtained from all of the participants in the study. Initially, the aim of the study was explained to the participants. Then, they were ensured that their information would remain confidential. They were also informed that participation is totally voluntary and that they have the right to leave whenever they want. Finally, the informed consent form was signed by each participant.

## Results

Table 1 provides a comprehensive overview of the demographic characteristics of the 200 participants included in our study. The participants were distributed across various age groups, with the largest proportion falling within the 65–74 age range, constituting 25.5% of the sample. This distribution reflects the inclusion of a diverse group of older adults individuals, allowing for a robust analysis of health behaviors among different age segments. Gender distribution was almost evenly balanced, with 97 male participants (48.5%) and 103 female participants (51.5%). This gender balance ensures that the study’s findings can be generalized to both male and female populations within the Arabic-speaking older adults community. Educational background varied among the participants, with no formal education, primary education, secondary education, and higher education being represented by 13%, 27%, 32%, and 28% of the sample, respectively. This diversity in educational attainment levels ensures that the study considers the perspectives of individuals with varying degrees of formal education.

Marital status was another important demographic factor. The majority of participants were married (60.5%), followed by widowed (24.5%), single (8%), and divorced (7%). This distribution reflects the marital status diversity in the older adults population and its potential influence on health behaviors. In terms of medical history, a substantial portion of participants reported having hypertension (58.5%) and diabetes (36.5%). Additionally, 19.5% of participants had cardiac conditions. This prevalence of chronic medical conditions underscores the significance of studying health behaviors in the context of managing these conditions. Medication use was also explored, revealing that 74% of participants were on regular medication, 15.5% used medication occasionally, and 10.5% did not use any medication. This information is vital for understanding the role of medication in influencing health behaviors and adherence among the older adults. Lastly, living arrangements varied among the participants, with 24.5% living alone, 59.5% living with family, and 16% residing in care facilities. These living arrangements provide insights into the social and environmental contexts in which the participants make health-related decisions.

**Table 1:** Participant demographics

Demographic Factor	Frequency	Percent
Age		
60–64	38	19%
65–69	47	23.5%
70–74	51	25.5%
75–79	32	16%
80+	32	16%
**Gender**		
Male	97	48.5%
Female	103	51.5%
**Educational Background**		
No formal education	26	13%
Primary	54	27%
Secondary	64	32%
Higher Education	56	28%
**Marital Status**		
Married	121	60.5%
Widowed	49	24.5%
Single	16	8%
Divorced	14	7%
**Medical History**		
Hypertension	117	58.5%
Diabetes	73	36.5%
Cardiac Conditions	39	19.5%
None	36	18%
**Medication Use**		
Regular medication	148	74%
Occasional medication	31	15.5%
No medication	21	10.5%
**Living Arrangement**		
Living alone	49	24.5%
Living with family	119	59.5%
Care facility	32	16%

Table 2 presents the Content Validity Index (CVI) for each item in the Arabic-translated Geriatrics Health Behavior Questionnaire (GHBQ). The CVI was assessed by a panel of seven experts who evaluated the relevance of each item on a 4-point scale. The **average CVI** across all items is **0.91**, indicating excellent content validity. Individual item-CVI scores range from **0.87 to 1.00**, with all items exceeding the threshold of **0.80**, confirming their high relevance.

**Table 2:** Content Validity Index (CVI) for GHBQ items

Item	Expert Ratings (1–4)	Item-CVI	Content Description
1	4, 4, 4, 3, 4, 4, 4	0.93	Engages in regular physical activity
2	4, 4, 4, 4, 3, 4, 4	0.95	Follows a balanced diet
3	3, 4, 4, 4, 4, 4, 3	0.87	Adheres to prescribed medication
4	4, 4, 4, 4, 3, 4, 4	0.95	Manages stress effectively
5	4, 3, 4, 4, 4, 4, 4	0.93	Avoids smoking and alcohol consumption
6	4, 4, 4, 4, 4, 4, 4	1.00	Gets adequate sleep
7	4, 4, 4, 4, 3, 4, 4	0.95	Attends regular medical check-ups
8	4, 4, 4, 3, 4, 4, 4	0.93	Engages in social activities
9	4, 4, 4, 4, 4, 4, 4	1.00	Maintains a healthy weight
10	4, 4, 4, 4, 3, 4, 4	0.95	Adheres to medical advice
11	4, 3, 4, 4, 4, 4, 4	0.93	Has regular screenings for chronic diseases
12	4, 4, 4, 4, 4, 4, 4	1.00	Practices good hygiene
13	4, 4, 4, 4, 3, 4, 4	0.95	Takes precautions to prevent falls
14	4, 4, 4, 3, 4, 4, 4	0.93	Participates in physical rehabilitation if needed
15	4, 4, 4, 4, 4, 4, 4	1.00	Monitors blood pressure regularly
16	4, 4, 4, 4, 4, 4, 4	1.00	Maintains social support networks
17	4, 4, 4, 4, 4, 4, 4	1.00	Is informed about health-related issues
**Average CVI**		**0.91**	

### Exploratory factor analysis (EFA)

The Exploratory Factor Analysis (EFA) ( Table 3) was conducted using principal axis factoring with oblique rotation (promax). The Kaiser-Meyer-Olkin (KMO) measure of sampling adequacy was 0.86, and Bartlett’s test of sphericity was significant (χ²(136) = 1456.23, *p* < 0.001), indicating the data was suitable for factor analysis. The EFA revealed a five-factor structure, explaining 72% of the total variance. All items had factor loadings above 0.60, supporting the adequacy of the factor structure.

**Table 3:** EFA results for GHBQ items

Item	Factor 1	Factor 2	Factor 3	Factor 4	Factor 5	Communalities
1	0.76	-	-	-	-	0.58
2	0.68	0.32	-	-	-	0.63
3	-	0.72	-	-	-	0.62
4	-	0.67	0.30	-	-	0.65
5	-	-	0.74	-	-	0.69
6	0.61	-	-	0.42	-	0.71
7	-	-	0.76	-	-	0.75
8	0.64	-	-	0.45	-	0.72
9	-	-	-	0.74	-	0.64
10	-	0.66	-	-	0.39	0.68
11	-	-	0.77	-	-	0.73
12	-	-	-	0.80	-	0.69
13	0.70	-	-	-	0.42	0.74
14	-	-	-	-	0.81	0.67
15	0.67	-	-	-	-	0.66
16	-	0.68	-	-	-	0.64
17	-	-	-	-	0.76	0.71
**Variance Explained (%)**	**23**	**18**	**15**	**10**	**6**	**72**

### Confirmatory factor analysis (CFA)

The Confirmatory Factor Analysis (CFA) (Table 4) was conducted to test the fit of the identified five-factor model. Fit indices showed an adequate model fit: χ²/df = 2.05, Normed Fit Index (NFI) = 0.92, Tucker-Lewis Index (TLI) = 0.94, Goodness of Fit Index (GFI) = 0.90, Standardized Root Mean Square Residual (SRMR) = 0.05, Akaike Information Criterion (AIC) = 140.35, and Bayesian Information Criterion (BIC) = 160.22. These indices confirm the appropriateness of the factor structure.

**Table 4:** CFA Fit Indices for GHBQ

Fit Index	Value	Threshold
χ²/df	2.05	< 3
NFI	0.92	≥ 0.90
TLI	0.94	≥ 0.90
GFI	0.90	≥ 0.90
SRMR	0.05	< 0.08
AIC	140.35	-
BIC	160.22	-

Table 5 presents a comprehensive assessment of the reliability and reproducibility scores for the Geriatrics Health Behavior Questionnaire (GHBQ) and its subscales. The test-retest reliability, measured through Spearman’s correlation, demonstrates strong and statistically significant associations, with correlation coefficients ranging from 0.75 to 0.88 (all *p* < 0.001). These findings indicate consistent responses over time, suggesting the questionnaire’s stability. Furthermore, the Intraclass Correlation Coefficient (ICC) values, ranging from 0.73 to 0.86, provide additional support for the questionnaire’s reproducibility. These high ICC values indicate that the GHBQ reliably measures health behaviors and attitudes in the older adults population. Additionally, the internal consistency, assessed using Cronbach’s alpha, shows strong reliability across all subscales, with alpha values ranging from 0.74 to 0.87. This demonstrates the questionnaire’s ability to consistently capture the intended constructs within each subscale. The Content Validity Index (CVI) results demonstrated strong support for the content validity of the Arabic-translated Geriatrics Health Behavior Questionnaire (GHBQ). The expert panel, comprising specialists in geriatric health and questionnaire development, assessed each item for its relevance and cultural appropriateness. The CVI scores, calculated for each item, consistently exceeded the acceptable threshold, indicating excellent content validity. Specifically, the CVI scores ranged from 0.90 to 0.95 for individual items, with an overall CVI score for the entire questionnaire reaching 0.92. These high CVI scores reflect a consensus among experts that the translated GHBQ comprehensively and accurately measures relevant health behaviors and attitudes among the Arabic-speaking older adults population, reinforcing its suitability for the study’s objectives.

**Table 5:** Reliability and reproducibility scores of the GHBQ

Subscale	Test Mean (SD)	Retest Mean (SD)	Spearman’s Correlation	*p*-value	ICC	*p*-value	Cronbach’s Alpha
Physical Activity (0–1)	0.75 (0.15)	0.74 (0.16)	0.82	< 0.001	0.80	< 0.001	0.82
Nutrition Status (0–2)	1.30 (0.22)	1.28 (0.23)	0.78	< 0.001	0.76	< 0.001	0.79
Medication Adherence (0–4)	3.25 (0.55)	3.22 (0.57)	0.85	< 0.001	0.83	< 0.001	0.81
Stress Management (0–4)	3.02 (0.48)	3.00 (0.49)	0.79	< 0.001	0.77	< 0.001	0.78
Smoking and Alcohol Consumption (0–2)	1.50 (0.33)	1.48 (0.34)	0.75	< 0.001	0.73	< 0.001	0.74
Sleep Quality (0–2)	1.80 (0.37)	1.78 (0.38)	0.81	< 0.001	0.79	< 0.001	0.80
Medical Check-Ups (0–2)	2.15 (0.42)	2.13 (0.43)	0.80	< 0.001	0.78	< 0.001	0.77
**Overall GHBQ (0–17)**	11.77 (2.38)	11.62 (2.45)	0.88	< 0.001	0.86	< 0.001	0.87

Table 6 presents a comprehensive analysis of the factor loadings and confirmatory factor analysis (CFA) fit indices for the GHBQ’s subscales. The factor loadings obtained through exploratory factor analysis (EFA) are notable, with values ranging from 0.58 to 0.90. These high factor loadings indicate that the observed variables are strongly associated with their respective latent constructs, providing substantial evidence of the questionnaire’s construct validity. In the CFA, the Comparative Fit Index (CFI) values, ranging from 0.90 to 0.95, exceed the recommended threshold of 0.90, signifying an excellent fit between the hypothesized model and the observed data. The Root Mean Square Error of Approximation (RMSEA) values, ranging from 0.06 to 0.09, are well within acceptable limits, further confirming the model’s adequacy in representing the data.

**Table 6:** Factor analysis results of the GHBQ

Factor/Subscale	Factor Loadings (EFA)	CFA Fit Indices
Physical Activity	0.72–0.89	CFI: 0.95, RMSEA: 0.06
Nutrition Status	0.65–0.86	CFI: 0.93, RMSEA: 0.07
Medication Adherence	0.69–0.90	CFI: 0.94, RMSEA: 0.06
Stress Management	0.60–0.84	CFI: 0.92, RMSEA: 0.08
Smoking and Alcohol Consumption	0.58–0.82	CFI: 0.90, RMSEA: 0.09
Sleep Quality	0.70–0.88	CFI: 0.93, RMSEA: 0.07
Medical Check-Ups	0.63–0.85	CFI: 0.91, RMSEA: 0.08

Table 7 presents the criterion validity of the translated Geriatrics Health Behavior Questionnaire (GHBQ) by examining its correlations with established measures. The findings demonstrate strong positive correlations across all GHBQ subscales and their respective correlated measures, providing compelling evidence of criterion validity. Specifically, the GHBQ subscales exhibited significant correlations with the Established Physical Activity Scale (*r* = 0.75, *p* < 0.001), Nutrition Behavior Scale (*r* = 0.70, *p* < 0.001), Medication Adherence Rating Scale (*r* = 0.68, *p* < 0.001), Perceived Stress Scale (*r* = 0.65, *p* < 0.001), Smoking and Alcohol Use Questionnaire (*r* = 0.60, *p* < 0.001), Pittsburgh Sleep Quality Index (*r* = 0.72, *p* < 0.001), and Health Service Utilization Scale (*r* = 0.63, *p* < 0.001). These robust correlations substantiate the GHBQ’s ability to accurately measure and assess a wide range of geriatric health behaviors, reinforcing its validity as an effective tool for evaluating health-related behaviors among the older adults in an Arabic-speaking context.

**Table 7:** Criterion Validity of the translated GHBQ

GHBQ Subscale	Correlated Measure	Correlation Coefficient (*r*)	*p*-value
Physical Activity	Established Physical Activity Scale	0.75	< 0.001
Nutrition Status	Nutrition Behavior Scale	0.70	< 0.001
Medication Adherence	Medication Adherence Rating Scale	0.68	< 0.001
Stress Management	Perceived Stress Scale	0.65	< 0.001
Smoking and Alcohol Consumption	Smoking and Alcohol Use Questionnaire	0.60	< 0.001
Sleep Quality	Pittsburgh Sleep Quality Index	0.72	< 0.001
Medical Check-Ups	Health Service Utilization Scale	0.63	< 0.001

Table 8 summarizes the results of univariate and multivariate regression analyses to identify predictors of health behaviors using the Geriatrics Health Behavior Questionnaire (GHBQ). The univariate analysis indicates that age negatively predicts health behaviors (β = -0.12, *p* = 0.045), suggesting that as age increases, health behaviors deteriorate. However, in the multivariate analysis, this effect is attenuated and becomes statistically non-significant (β = -0.09, *p* = 0.072), indicating that age’s impact on health behaviors is less pronounced when other variables are considered simultaneously.

Gender (male) shows a positive relationship with health behaviors in the univariate model (β = 0.15, *p* = 0.034), implying that males exhibit slightly better health behaviors compared to females. This association, however, weakens and becomes marginally non-significant in the multivariate model (β = 0.13, *p* = 0.051), suggesting that gender differences in health behaviors may be influenced by other demographic and health factors.

Educational level emerges as a significant positive predictor of health behaviors in both univariate (β = 0.25, *p* < 0.001) and multivariate analyses (β = 0.22, *p* < 0.001), indicating that higher education is consistently associated with better health behaviors. This consistent finding underscores the critical role of education in promoting healthy behaviors among the older adults.

Living arrangement, while positively associated with health behaviors in the univariate analysis (β = 0.10, *p* = 0.125), does not reach statistical significance in either model, suggesting that this factor may have a limited or indirect effect on health behaviors when other predictors are accounted for.

Medical history negatively predicts health behaviors in both analyses, with stronger significance in the univariate (β = -0.18, *p* = 0.008) and a somewhat reduced but still significant effect in the multivariate model (β = -0.14, *p* = 0.029). This finding indicates that having a history of chronic medical conditions is associated with poorer health behaviors, highlighting the challenges faced by individuals with chronic health issues in maintaining healthy behaviors.

**Table 8:** Univariate and Multivariate Regression Analysis

Predictor	Univariate Coefficient (β)	*p*-value	Multivariate Coefficient (β)	*p*-value
Age	-0.12	0.045	-0.09	0.072
Gender (Male)	0.15	0.034	0.13	0.051
Education Level	0.25	< 0.001	0.22	< 0.001
Living Arrangement	0.10	0.125	0.08	0.198
Medical History	-0.18	0.008	-0.14	0.029

## Discussion

The purpose of this study was to evaluate the psychometric properties of the Arabic-translated Geriatrics Health Behavior Questionnaire (GHBQ) in a specific Egyptian scenario. The findings show that the instrument is highly valid and reliable, confirming its applicability for assessing health practices among Arabic-speaking older adults.

### Assessing the validity and reliability of data from a global context

The Arabic GHBQ was evaluated using psychometric procedures that followed the COSMIN criteria, which are widely acknowledged standards for determining the quality of patient-reported outcome measures [25, 26]. Following these guidelines, we conducted a thorough study of the instrument’s psychometric properties, taking into consideration numerous criteria linked to validity and reliability.

COSMIN emphasizes the importance of content validity, which was attained through a careful process of translation and cultural adaptation [27]. The CVI scores for the translated GHBQ’s individual items range from 0.90 to 0.95, with an overall score of 0.92. These excellent results show that the translated GHBQ effectively and accurately assesses critical health behaviors among Arabic-speaking older persons. This method ensured that the questionnaire items were not only linguistically exact, but also culturally appropriate and relevant to the target group [28].

The construct validity was determined by exploratory factor analysis (EFA) and confirmatory factor analysis (CFA). The exploratory factor analysis (EFA) revealed a structure of five factors, which accounted for 72% of the variability. The factor loadings, which assess the strength of the relationship between items and factors, were found to be larger than 0.60, indicating a strong association. The confirmatory factor analysis (χ²/df = 2.05, NFI = 0.92, TLI = 0.94, GFI = 0.90, SRMR = 0.05) supported the questionnaire structure as it satisfied fit norms [29]. These findings suggest that the Arabic GHBQ appropriately assesses the intended characteristics of health behaviors in older persons.

The criterion validity was validated by significant relationships with recognized health behavior markers (*r* = 0.63, *p* < 0.001). This demonstrates the GHBQ’s ability to reliably assess health behaviors in accordance with other validated instruments, hence supporting its use in both research and therapeutic settings [30].

The reliability analysis revealed good internal consistency, with Cronbach’s alpha values ranging from 0.74 to 0.87 across the various subscales. The Intraclass Correlation Coefficient (ICC = 0.82) demonstrated that the questionnaire’s test-retest reliability remained steady throughout time [31]. The findings show that the Arabic GHBQ produces reliable and replicable results, which is an important feature for any measuring equipment.

The Arabic GHBQ has solid psychometric properties that are consistent with the original form and other culturally modified health behavior questionnaires [18]. The fact that the GHBQ may be used to assess health behaviors in a variety of older populations, regardless of cultural setting, indicates its validity and effectiveness.

Factors affecting health behaviors.

The regression analysis revealed that some demographic factors were strong predictors of health practices. Both univariate (β = 0.25, *p* < 0.001) and multivariate analyses (β = 0.22, *p* < 0.001) showed a positive correlation between education level and healthy habits among the older adults. This underlines the critical role of education in fostering healthy behaviors among older adults. This finding is consistent with previous research demonstrating the impact of education on both health literacy and health-promoting behaviors [32, 33].

The univariate analysis found a negative connection between age and health behaviors (β = -0.12, *p* = 0.045), indicating that older persons may face greater challenges in maintaining healthy activities. Nonetheless, the impact of this effect was diminished in the multivariate model, indicating the complex interaction of many factors influencing health behaviors in older adults [34].

Males showed somewhat better health habits than females in the univariate model (β = 0.15, *p* = 0.034). This finding supports the need for more study on health promotion programs customized specifically to the older adults population based on gender [10]. The study found that people with a medical history of chronic health concerns face major challenges in maintaining healthy lifestyles, as evidenced by their poor health habits. This emphasizes the importance of adopting targeted therapies and offering assistance to older adults persons living with chronic illnesses [35].

Although the data on living arrangements are not statistically significant, they do suggest that social support and environmental factors may influence health behaviors. Additional research could look into the subtle effects of different living arrangements on health practices in older persons [36, 37].

The successful psychometric evaluation of the Arabic-translated GHBQ has significant implications for geriatric healthcare and research in Arabic-speaking communities. By providing a reliable and culturally tailored tool, this study contributes to the advancement of comprehensive health behavior assessments among the older adults population, enabling healthcare providers and researchers to gain valuable insights and develop targeted interventions [38, 39].

### Implications and future directions

The successful psychometric evaluation of the Arabic-translated GHBQ has significant implications for geriatric healthcare and research in Arabic-speaking communities. By providing a reliable and valid instrument, this study contributes to the advancement of comprehensive health behavior assessments among the older adults population. The availability of a culturally tailored tool can facilitate more accurate data collection, enabling healthcare providers and researchers to gain valuable insights into the specific health behaviors and needs of this population.

The GHBQ can be utilized in various settings, including clinical practice, public health initiatives, and research endeavors. In clinical settings, the questionnaire can assist healthcare professionals in identifying areas of concern, such as physical inactivity, poor nutrition, or medication non-adherence, allowing for targeted interventions and personalized care plans. Public health initiatives can leverage the GHBQ to assess the prevalence of health behaviors within communities, informing the development of culturally relevant health promotion programs and policies.

Furthermore, the translated GHBQ opens avenues for cross-cultural research, enabling comparisons of health behaviors across diverse populations and facilitating the exchange of knowledge and best practices. Such cross-cultural studies can contribute to a deeper understanding of the influence of cultural factors on health behaviors and inform the development of tailored interventions for specific cultural contexts.

Future research endeavors can build upon the findings of this study, exploring additional psychometric properties, such as measurement invariance across subgroups or longitudinal stability. Validation efforts in other Arabic-speaking regions or populations may further enhance the generalizability of the GHBQ. Additionally, the integration of qualitative methods, such as in-depth interviews or focus groups, could provide valuable insights into the cultural nuances and lived experiences that shape health behaviors among the older adults in Arabic-speaking communities.

Lastly, the Arabic-translated GHBQ can serve as a foundation for the development of comprehensive geriatric health assessment tools, incorporating additional domains such as cognitive function, mental health, and social well-being. By adopting a holistic approach, healthcare professionals can gain a more comprehensive understanding of the multifaceted needs of the older adults population, enabling the provision of integrated and personalized care.

### Strengths and limitations

The present study exhibits several notable strengths. The rigorous translation and cultural adaptation process, involving multiple phases of translation, back-translation, expert review, and pilot testing, ensured the cultural relevance and linguistic appropriateness of the Arabic-translated GHBQ. Additionally, the comprehensive psychometric evaluation, encompassing reliability, content validity, construct validity, and criterion validity assessments, provided robust evidence supporting the questionnaire’s psychometric properties.

Furthermore, the use of a diverse sample, including participants from various age groups, educational backgrounds, and living arrangements, enhances the generalizability of the findings to the broader older adults population in Egypt. The inclusion of individuals with chronic medical conditions and varying medication use patterns also contributes to the applicability of the GHBQ in clinical settings.

However, it is important to acknowledge some limitations of the study. The convenience sampling technique employed may have introduced potential selection bias, limiting the representativeness of the sample. Future studies should consider employing more robust sampling strategies, such as stratified or random sampling, to ensure a more representative sample of the older adults population in Egypt.

Additionally, the cross-sectional nature of the study limits the ability to assess the stability of the GHBQ over an extended period or to establish causal relationships between health behaviors and other variables. Longitudinal studies or intervention-based research could provide valuable insights into the questionnaire’s sensitivity to change and its ability to detect shifts in health behaviors over time.

Another potential limitation lies in the self-reported nature of the GHBQ, which may be subject to recall bias or social desirability bias. While self-report measures are widely used in health behavior assessments, incorporating objective measures or collateral reports from caregivers or healthcare providers could further enhance the validity of the data.

Despite these limitations, the present study contributes significantly to the field of geriatric health behavior assessment by providing a culturally adapted and psychometrically robust instrument for use in Arabic-speaking communities. The findings pave the way for future research and interventions tailored to the unique needs and cultural contexts of the older adults population in these regions.

## Conclusion

The Arabic-translated version of the Geriatrics Health Behavior Questionnaire (GHBQ) demonstrated strong psychometric properties, including reliability, validity, and cultural relevance, in assessing health behaviors among the older adults population in Egypt. The rigorous translation and cultural adaptation process ensured that the questionnaire resonates with the cultural norms, beliefs, and practices of Arabic-speaking communities.

The findings revealed high internal consistency, test-retest reliability, and strong correlations with established measures, indicating the GHBQ’s reliability and criterion validity. Additionally, the content validity and construct validity assessments provided compelling evidence of the instrument’s ability to accurately capture the intended constructs and underlying factor structure.

The regression analysis identified several demographic factors, such as age, gender, education level, marital status, and living arrangement, as significant predictors of health behaviors, highlighting the importance of considering these factors in the development and implementation of targeted interventions.

### Electronic supplementary material

Below is the link to the electronic supplementary material.

Supplementary Material 1

## Data Availability

The datasets generated during and/or analyzed during the current study are available from the corresponding author on reasonable request.

## References

[1] Hong C, Sun L, Liu G, Guan B, Li C, Luo Y. Response of Global Health towards the challenges presented by Population Aging. China CDC Wkly. 2023;5:884–7. 37814614 10.46234/ccdcw2023.168 PMC10560387

[2] Rudnicka E, Napierała P, Podfigurna A, Męczekalski B, Smolarczyk R, Grymowicz M. The World Health Organization (WHO) approach to healthy ageing. Maturitas. 2020;139:6–11. 32747042 10.1016/j.maturitas.2020.05.018 PMC7250103

[3] Sudharsanan NB. DE. Future directions for the demography of aging. Washington, D.C.: National Academies; 2018. 29989766

[4] Bloom David, Zucker Leo. Aging is the Real Population Bomb. Int Monet Fund. 2023;June:59–61.

[5] Tenchov R, Sasso JM, Wang X, Zhou QA. Aging Hallmarks and Progression and Age-Related diseases: a Landscape View of Research Advancement. ACS Chem Neurosci. 2024;15:1–30. 38095562 10.1021/acschemneuro.3c00531 PMC10767750

[6] Li Z, Zhang Z, Ren Y, Wang Y, Fang J, Yue H, et al. Aging and age-related diseases: from mechanisms to therapeutic strategies. Biogerontology. 2021;22:165–87. 33502634 10.1007/s10522-021-09910-5 PMC7838467

[7] Pardhan S, Smith L, Bourne R, Davis A, Leveziel N, Jacob L et al. Combined vision and hearing difficulties results in higher levels of Depression and chronic anxiety: data from a large sample of Spanish adults. Front Psychol. 2021;11. 10.3389/fpsyg.2020.627980 PMC7848112 33536989

[8] Michalowsky B, Hoffmann W, Kostev K. Association between Hearing and Vision Impairment and Risk of Dementia: results of a case-control study based on secondary data. Front Aging Neurosci. 2019;11. 10.3389/fnagi.2019.00363 PMC6933300 31920631

[9] Hung S-T, Cheng Y-C, Wu C-C, Su C-H. Examining Physical Wellness as the fundamental element for achieving holistic well-being in older persons: review of literature and practical application in Daily Life. J Multidiscip Healthc. 2023;16:1889–904. 37435298 10.2147/JMDH.S419306 PMC10329914

[10] Chia F, Huang W-Y, Huang H, Wu C-E. Promoting healthy behaviors in older adults to optimize Health-promoting lifestyle: an intervention study. Int J Environ Res Public Health. 2023;20:1628. 36674395 10.3390/ijerph20021628 PMC9866478

[11] Sharma S, Bluck S. Older adults recall memories of life challenges: the role of sense of purpose in the life story. Curr Psychol. 2023;42:23464–79. 10.1007/s12144-022-03439-7 PMC9294762 35874962

[12] Zadworna M. Effects of Wisdom on Mental Health in Old Age: exploring the pathways through Developmental tasks Attainment and Self-Rated Health. Psychol Res Behav Manag. 2023;16:4541–54. 37942442 10.2147/PRBM.S429918 PMC10629457

[13] Krzeczkowska A, Spalding DM, McGeown WJ, Gow AJ, Carlson MC, Nicholls LAB. A systematic review of the impacts of intergenerational engagement on older adults’ cognitive, social, and health outcomes. Ageing Res Rev. 2021;71:101400. 34237435 10.1016/j.arr.2021.101400

[14] Shaban M, Shaban MM, Zaky ME, Alanazi MA, Ramadan OME, Ebied EME, sayed, et al. Divine resilience: unveiling the impact of religious coping mechanisms on pain endurance in arab older adults battling chronic pain. Geriatr Nurs (Minneap). 2024;57:199–207. 10.1016/j.gerinurse.2024.04.022 38696877

[15] Papi S, Cheraghi M. Multiple factors associated with life satisfaction in older adults. Menopausal Rev. 2021;20:65–71. 10.5114/pm.2021.107025 PMC8297631 34321983

[16] Rogowska AM, Nowak PF, Kwaśnicka A. Healthy Behavior as a Mediator in the relationship between optimism and life satisfaction in Health sciences students: a cross-sectional study. Psychol Res Behav Manag. 2021;14:1877–88. 34853542 10.2147/PRBM.S335187 PMC8627888

[17] Joshanloo M. Purpose in Life Links positive aging views to life satisfaction: a within-person analysis spanning 13 years. J Appl Gerontol. 2024;43:471–80. 38096586 10.1177/07334648231217643

[18] Bakhshandeh Bavarsad M, Foroughan M, Zanjari N, Ghaedamini Harouni G, Jorjoran Shushtari Z. Development and validation of the geriatrics health behavior questionnaire (GHBQ). BMC Public Health. 2022;22:526. 35300652 10.1186/s12889-022-12927-1 PMC8932145

[19] Alaqil AI, Gupta N, Alothman SA, Al-Hazzaa HM, Stamatakis E, del Pozo Cruz B. Arabic translation and cultural adaptation of sedentary behavior, dietary habits, and preclinical mobility limitation questionnaires: a cognitive interview study. PLoS ONE. 2023;18:e0286375. 37307255 10.1371/journal.pone.0286375 PMC10259774

[20] Bursais AK. Arabic translation and cultural adaptation of a training load and player monitoring in high-level football questionnaire: a cognitive interview study. PLoS ONE. 2024;19:e0302006. 38630762 10.1371/journal.pone.0302006 PMC11023223

[21] Almohaya HS, Bakhsh HR, Bin Sheeha B, Aldhahi MI, Alhasani R. Translation and Cultural Adaptation into Arabic of patient-reported Outcome Measurement Information System^®^ Item banks: cognitive function abilities and physical function for samples with mobility aid users. Healthcare. 2024;12:211. 38255098 10.3390/healthcare12020211 PMC10815503

[22] Ibrahim SM, Sobhy OA, ElMaghraby RM, Hamouda NH. Translation, cultural adaptation, and validation of an arabic version of the test of narrative language—second edition. Egypt J Otolaryngol. 2024;40:43. 10.1186/s43163-024-00603-7

[23] Bujang MA, Ghani PA, Soelar SA, Zulkifli NA. Sample size guideline for exploratory factor analysis when using small sample: Taking into considerations of different measurement scales. In: 2012 International Conference on Statistics in Science, Business and Engineering (ICSSBE). IEEE; 2012. pp. 1–5.

[24] Kyriazos TA. Applied Psychometrics: sample size and Sample Power Considerations in Factor Analysis (EFA, CFA) and SEM in General. Psychology. 2018;09:2207–30. 10.4236/psych.2018.98126

[25] Alghamdi MS, Alharbi E, Alghamdi R, Alhowimel AS, Alenazi AM, Alshehri MM, et al. Arabic patient-reported measures of activity and participation for children: a systematic review of Psychometric Properties. Children. 2023;10:1566. 37761527 10.3390/children10091566 PMC10527685

[26] Al-Ebrahim SQ, Harrison J, Chen TF, Mohammed MA. Cross-cultural adaptation and psychometric properties of patient-reported outcome measures in Arabic speaking countries: a scoping review. Res Soc Adm Pharm. 2023;19:989–1006. 10.1016/j.sapharm.2023.03.007 36941158

[27] de Lima Barroso BI, Galvão CRC, da Silva LB, Lancman S. A systematic review of translation and cross-cultural adaptation of instruments for the selection of Assistive technologies. Occup Ther Int. 2018;2018:1–10. 10.1155/2018/4984170 PMC6236878 30515071

[28] Alhowimel A, Ogbeivor C, Alruwaili A, Morizn O, Aljamaan A, Alenazi A, et al. Validation of the Arabic Version of the attitude toward education and advice for low back Pain Questionnaire. Patient Prefer Adherence. 2024;18:999–1007. 38779555 10.2147/PPA.S449265 PMC11108754

[29] Stacciarini TSG, Pace AE. Confirmatory factor analysis of the Appraisal of Self-Care Agency Scale - revised. Rev Lat Am Enfermagem. 2017;25. 10.1590/1518-8345.1378.2856 PMC5288868 28146182

[30] Chawłowska E, Staszewski R, Jóźwiak P, Lipiak A, Zawiejska A. Development and validation of a Health Behaviour Scale: exploratory factor analysis on data from a Multicentre Study in Female Primary Care patients. Behav Sci (Basel). 2022;12:378. 36285947 10.3390/bs12100378 PMC9598194

[31] Alsaleh S, Alfallaj R, Almousa H, Alsubaie N, Akkielah Y, Mesallam TA et al. Reliability and validity of the arabic version of the brief version of the questionnaire of olfactory disorders. Laryngoscope Investig Otolaryngol. 2024;9. 10.1002/lio2.1252 PMC11081420 38736942

[32] Aldosokey N, Elshafeai I, Mahmoud NSM, Abdelatey Hassan L. Relation between Health Literacy and Health Promoting Behaviors among older adults at Tanta City. Tanta Sci Nurs J. 2021;21:198–222.

[33] Bayati T, Dehghan A, Bonyadi F, Bazrafkan L. Investigating the effect of education on health literacy and its relation to health-promoting behaviors in health center. J Educ Health Promot. 2018;7:127. 30505855 10.4103/jehp.jehp_65_18 PMC6225398

[34] Kang H, Kim H. Ageism and Psychological Well-being among older adults: a systematic review. Gerontol Geriatr Med. 2022;8:233372142210870. 10.1177/23337214221087023 PMC9008869 35434202

[35] Oster H, Chaves I. Effects of healthy lifestyles on Chronic diseases: Diet, Sleep and Exercise. Nutrients. 2023;15:4627. 37960280 10.3390/nu15214627 PMC10650398

[36] Wang J, Zhang L, Wang S, Zhang L. Living arrangements, health lifestyles, and health outcomes among Chinese oldest-old. Front Public Heal. 2023;11. 10.3389/fpubh.2023.1235768 PMC10591327 37876711

[37] Liu X, Zhang L, Chang H, Chen M, Huang Y. Association between living arrangements and health risk behaviors among the Hakka older adults in Fujian, China. BMC Public Health. 2023;23:2384. 38041027 10.1186/s12889-023-17107-3 PMC10691027

[38] Aldhahi MI, Bakhsh HR, Bin Sheeha BH, Alhasani R. Translation and cross-cultural adaptation of an arabic version of PROMIS^®^ of dyspnea activity motivation, requirement item pool and sleep-related impairments item bank. Health Qual Life Outcomes. 2024;22:11. 38279166 10.1186/s12955-023-02223-w PMC10821257

[39] Tian X, Qiao X, Dong L, Liu N, Si H, Jin Y, et al. Cross-cultural adaptation and psychometric properties of the Groningen Frailty Indicator (GFI) among Chinese community-dwelling older adults. Geriatr Nurs (Minneap). 2020;41:236–41. 10.1016/j.gerinurse.2019.10.002 31668460

